# Supplementary material for: Comparison between passive knee kinematics during surgery and active knee kinematics during walking: A preliminary study
Source: PLoS One. 2023 Mar 6;18(3):e0282517. doi: 10.1371/journal.pone.0282517 (PMC9987822; doi:10.1371/journal.pone.0282517)
Supplement: S1 File — (PDF) [file pone.0282517.s001.pdf]

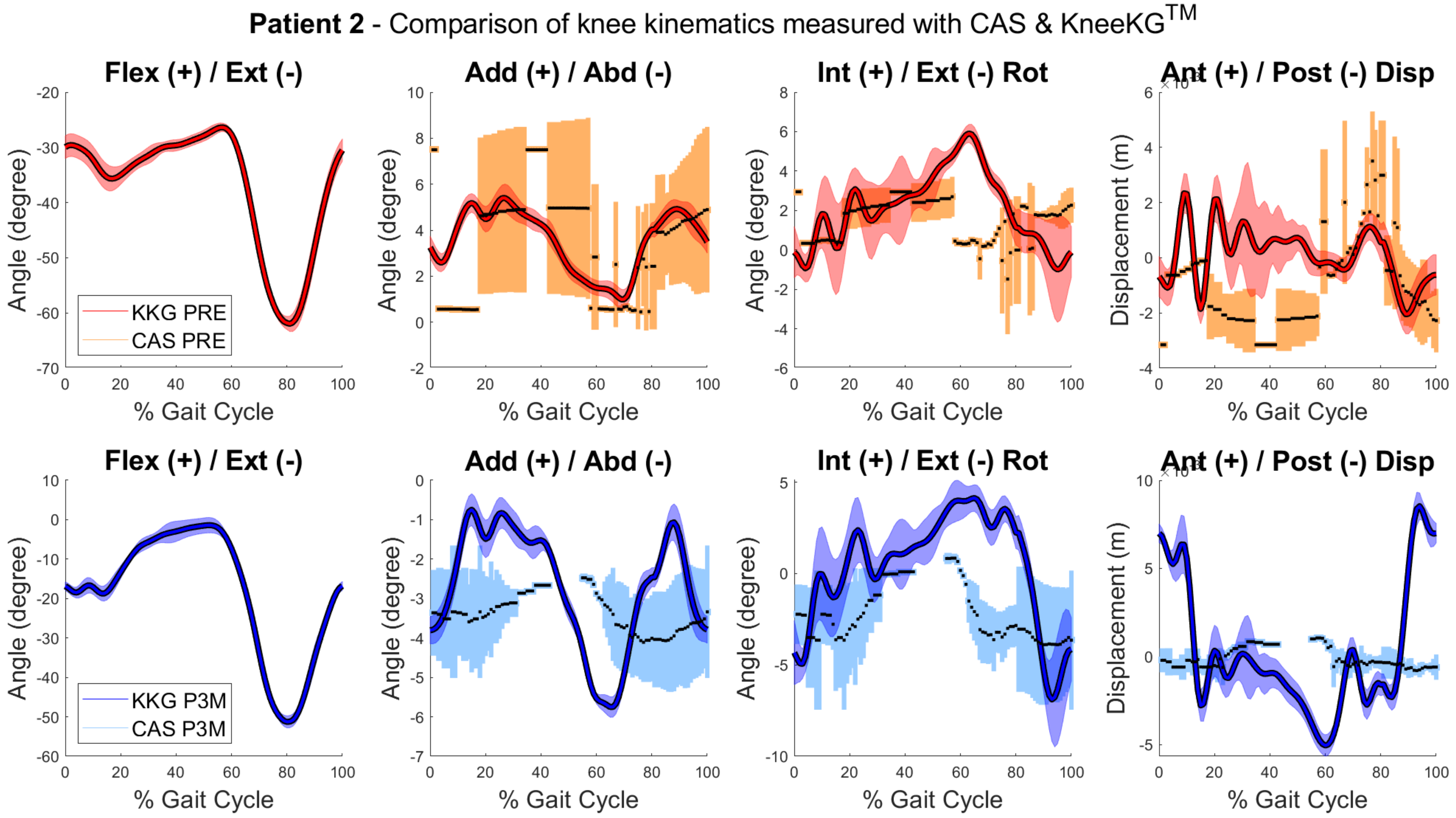
Patient 2 - Comparison of knee kinematics measured with CAS & KneeKG™
Flex (+) / Ext (-)
Add (+) / Abd (-)
Int (+) / Ext (-) Rot
Ant (+) / Post (-) Disp
Angle (degree)
Displacement (m)
×10⁻³
% Gait Cycle
KKG PRE
CAS PRE
KKG P3M
CAS P3M

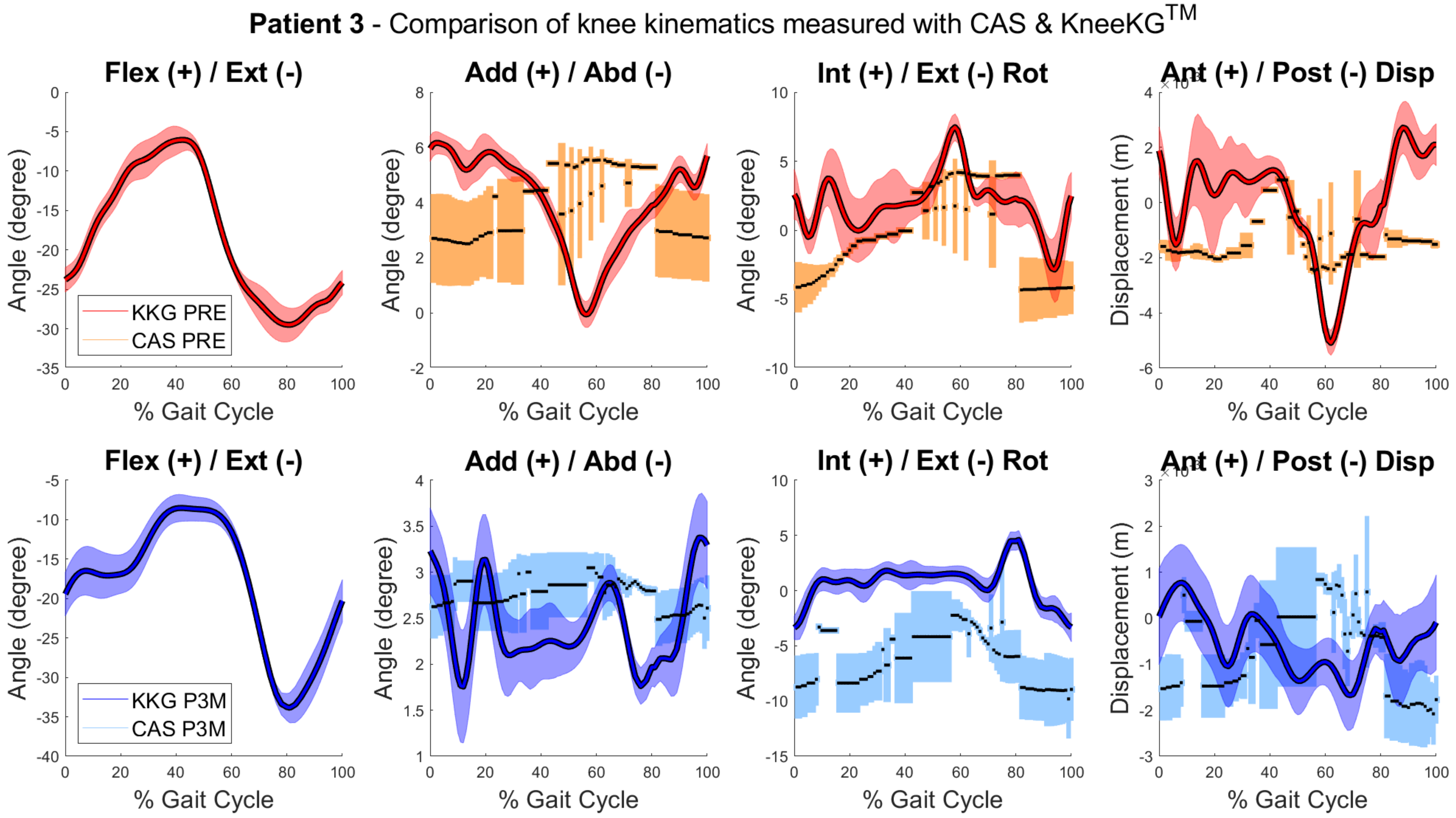

Patient 3 - Comparison of knee kinematics measured with CAS & KneeKG™
Flex (+) / Ext (-)
Add (+) / Abd (-)
Int (+) / Ext (-) Rot
Ant (+) / Post (-) Disp
Angle (degree)
Displacement (m)
% Gait Cycle
KKG PRE
CAS PRE
KKG P3M
CAS P3M

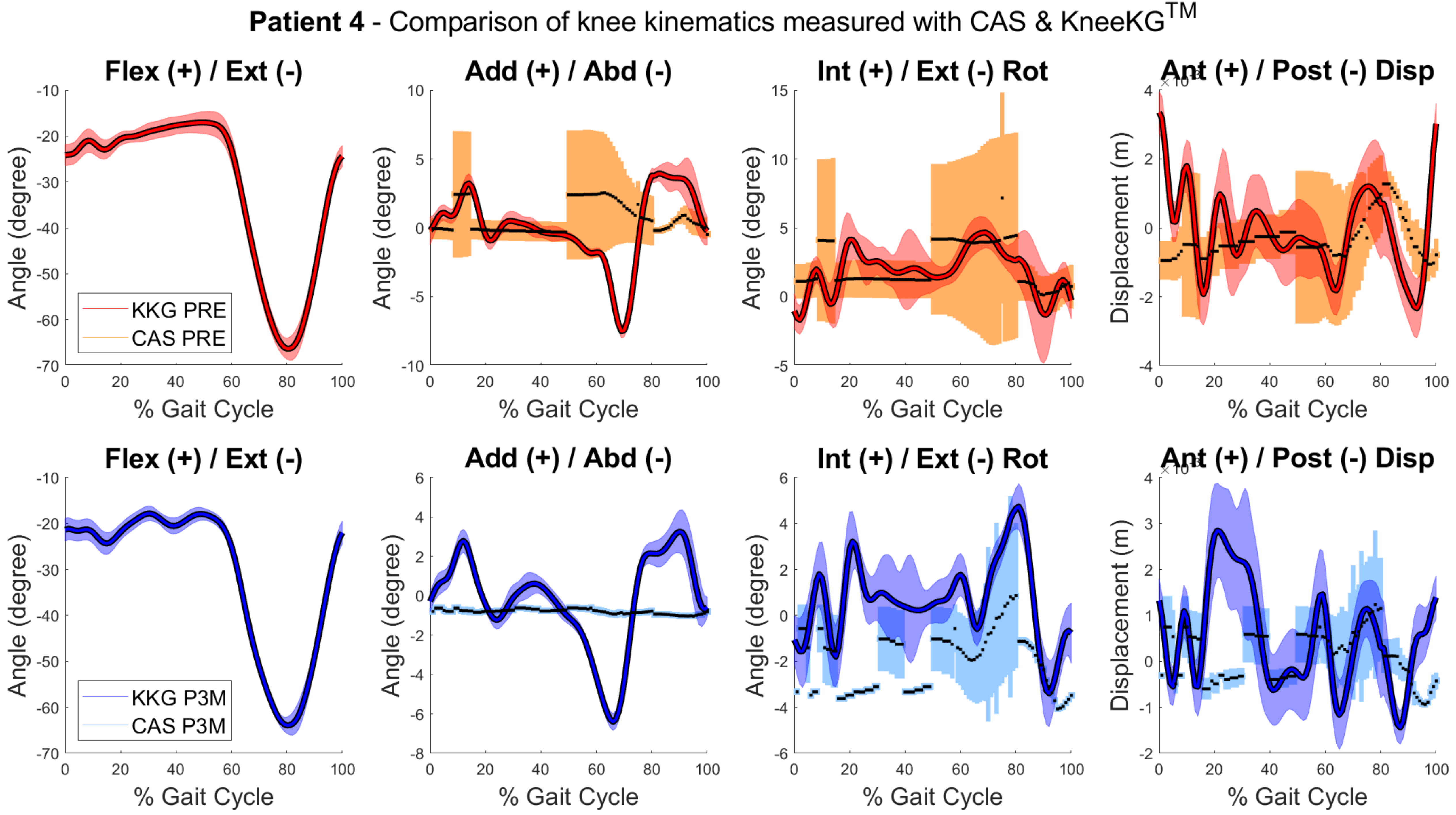

Patient 4 - Comparison of knee kinematics measured with CAS & KneeKG™
Flex (+) / Ext (-)
Add (+) / Abd (-)
Int (+) / Ext (-) Rot
Ant (+) / Post (-) Disp
Angle (degree)
Displacement (m)
% Gait Cycle
KKG PRE
CAS PRE
KKG P3M
CAS P3M

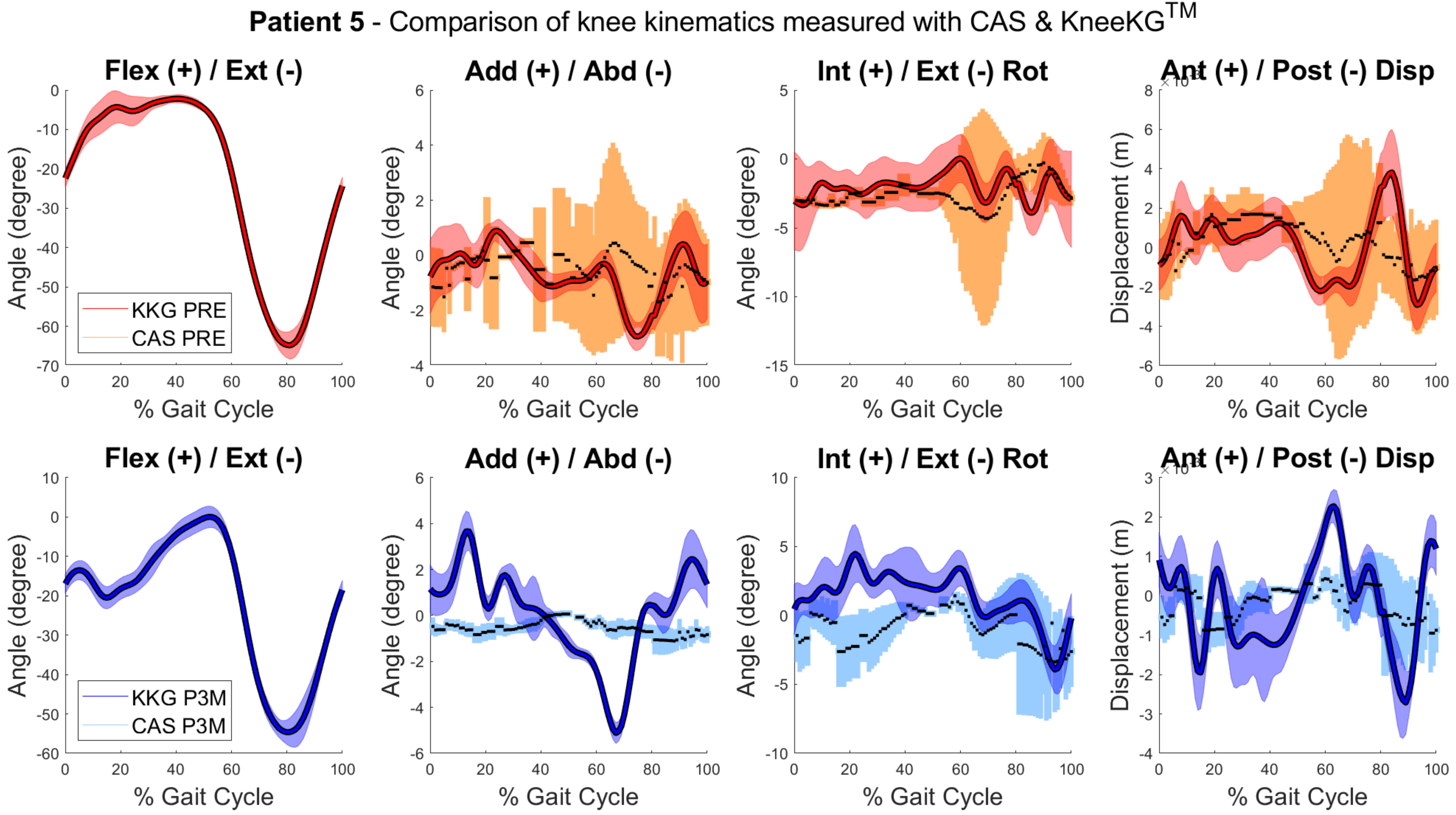

Patient 5 - Comparison of knee kinematics measured with CAS & KneeKG™
Flex (+) / Ext (-)
Add (+) / Abd (-)
Int (+) / Ext (-) Rot
Ant (+) / Post (-) Disp
Angle (degree)
Displacement (m)
×10⁻³
% Gait Cycle
KKG PRE
CAS PRE
KKG P3M
CAS P3M

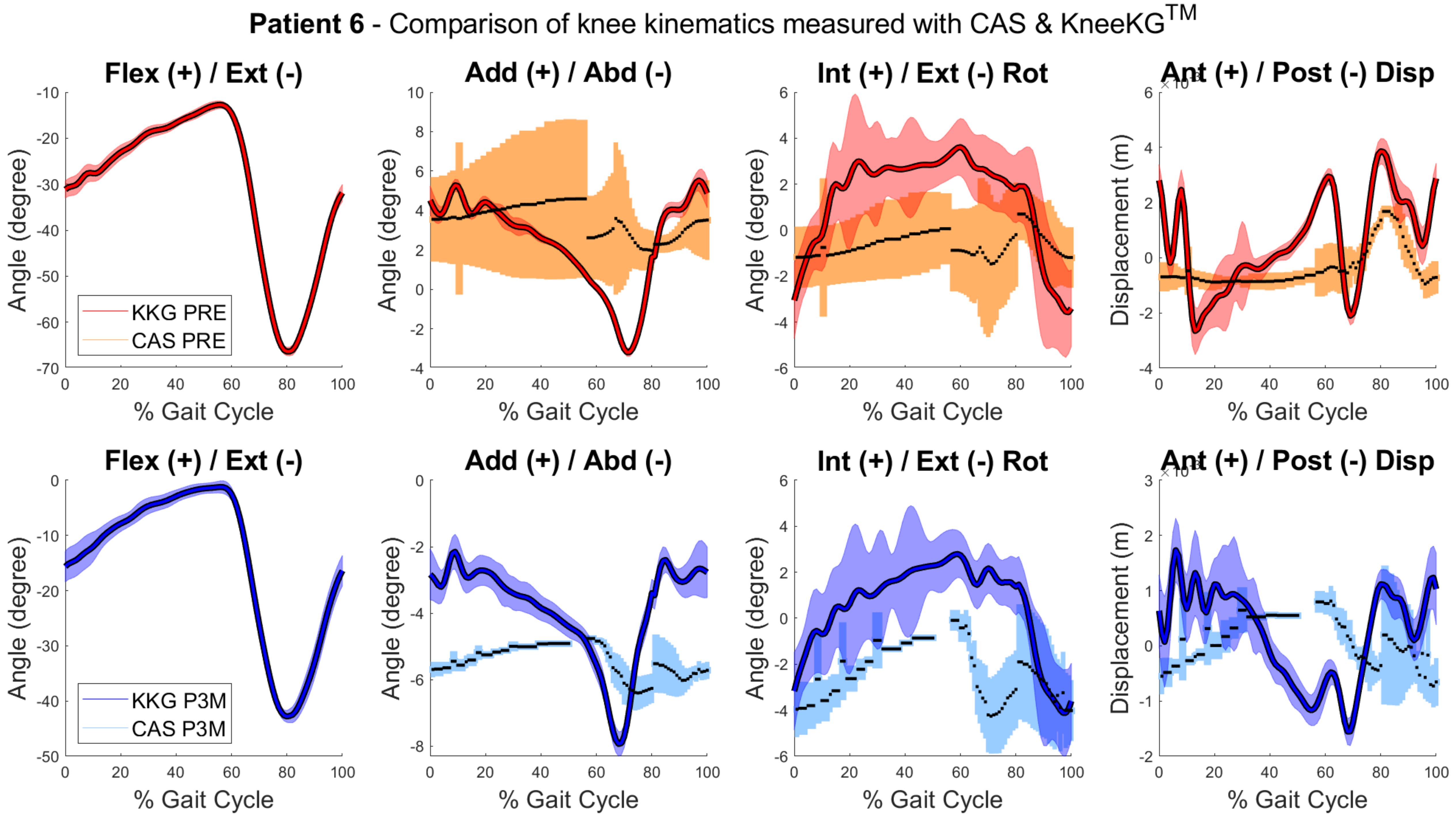
Patient 6 - Comparison of knee kinematics measured with CAS & KneeKG™
Flex (+) / Ext (-)
Add (+) / Abd (-)
Int (+) / Ext (-) Rot
Ant (+) / Post (-) Disp
Angle (degree)
Displacement (m)
×10⁻³
% Gait Cycle
KKG PRE
CAS PRE
KKG P3M
CAS P3M

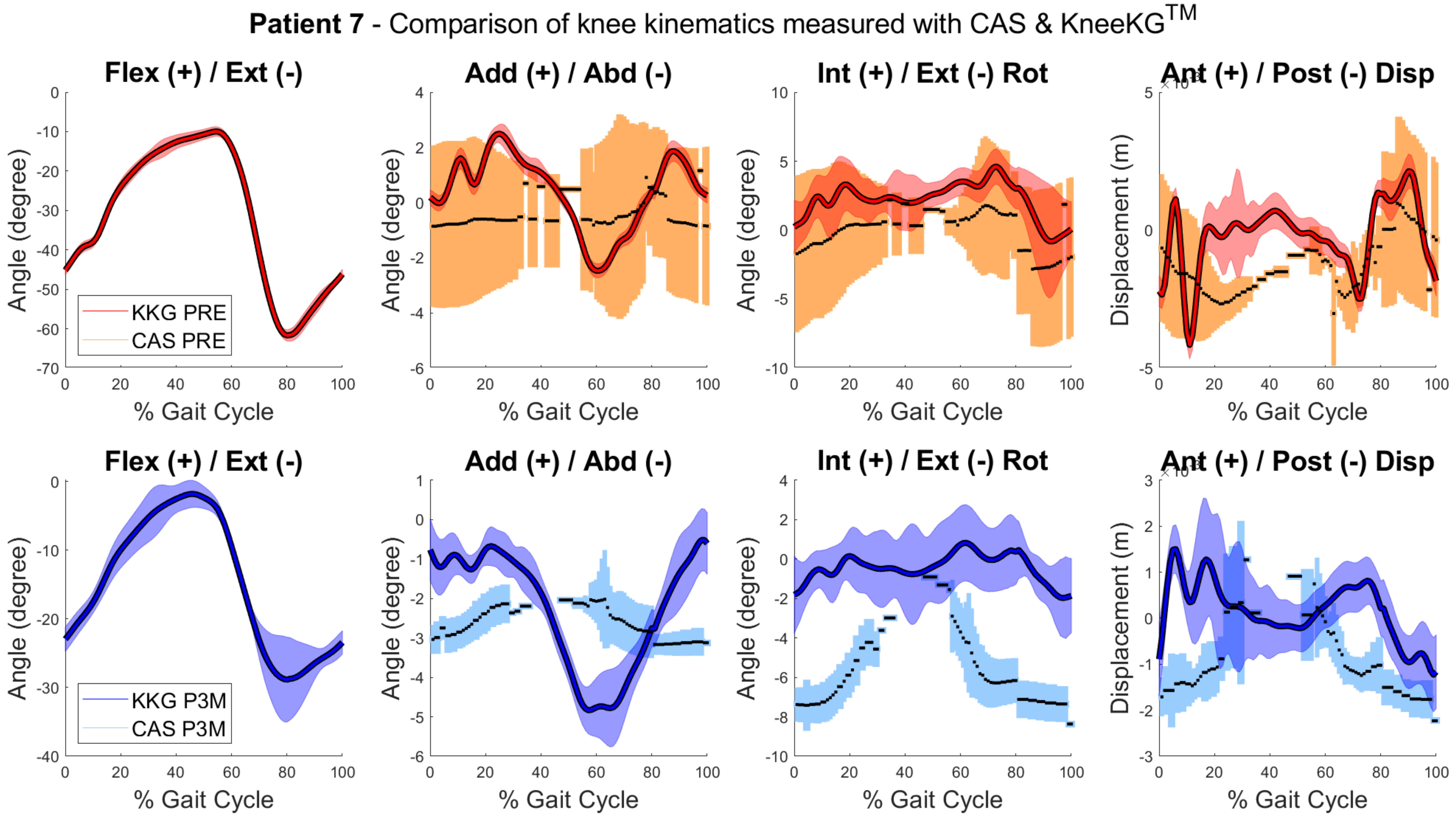
Patient 7 - Comparison of knee kinematics measured with CAS & KneeKG™
Flex (+) / Ext (-)
Add (+) / Abd (-)
Int (+) / Ext (-) Rot
Ant (+) / Post (-) Disp
Angle (degree)
Displacement (m)
% Gait Cycle
KKG PRE
CAS PRE
KKG P3M
CAS P3M

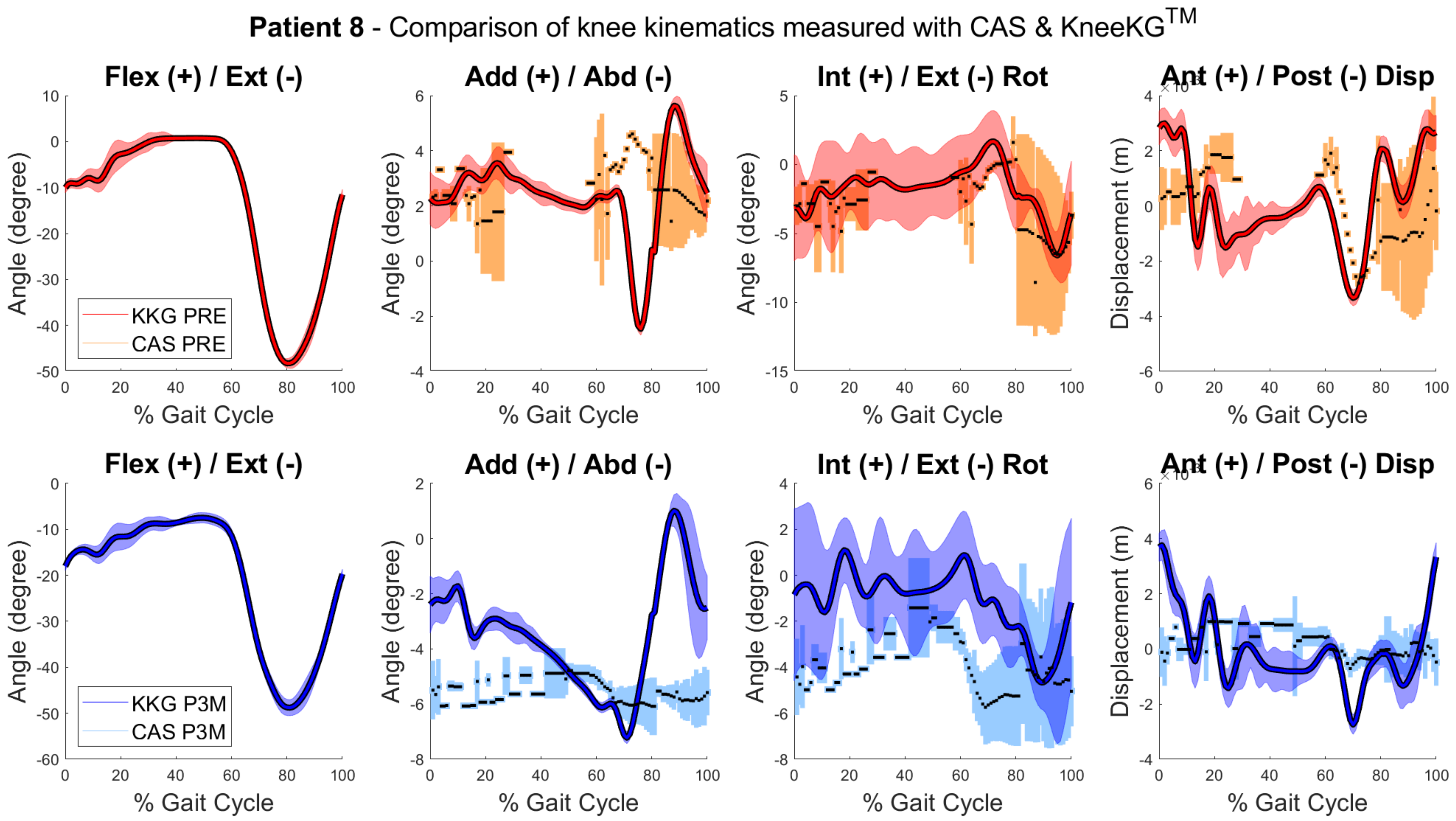
Patient 8 - Comparison of knee kinematics measured with CAS & KneeKG™
Flex (+) / Ext (-)
Add (+) / Abd (-)
Int (+) / Ext (-) Rot
Ant (+) / Post (-) Disp
Angle (degree)
Displacement (m)
×10⁻³
% Gait Cycle
KKG PRE
CAS PRE
KKG P3M
CAS P3M
