# Supplementary material for: Comparison between passive knee kinematics during surgery and active knee kinematics during walking: A preliminary study
Source: PLoS One. 2023 Mar 6;18(3):e0282517. doi: 10.1371/journal.pone.0282517 (PMC9987822; doi:10.1371/journal.pone.0282517)
Supplement: Attachment — Submitted filename: NK - Response to reviewers_Final.DOCX [file pone.0282517.s002.DOCX]

**Response to reviewers**

We would like to thank the editor and reviewers for their valuable comments that will help us improve the quality of the paper.

Reviewer #1: Thank you for reviewing this interesting study. Although the reviewer understands this is a preliminary study, the limited number of patients (n=8) cannot add any information about the comparison between passive knee kinematics during surgery and active knee kinematics during walking.

We agree the number of subjects is limited. That is why the title clearly states it is a preliminary study. Still, the authors think the difficulty to compare passive and active knee kinematics in a clinical environment (i.e. without relying on fluoroscopy but on generally available navigation and motion capture) comes more from the measurements (bone vs. skin) the than the number of subjects.

We do think that as pointed out by reviewer #2, “this is appropriate for a preliminary study such as this one.”

Moreover, the contribution of this study is also methodological as big efforts have been made to match coordinates systems and joint angle outcomes. This is largely addressed in the discussion and was pointed out by reviewer 2 “the value in this manuscript to readers is in understanding the methods the authors have used to relate the two distinctly derived knee kinematic measures.”

Reviewer #2: This study aims to develop a relationship between passive knee kinematics obtained during surgery and active kinematics during gait following surgery. This is an interesting idea, which immediately brings several concerns to mind, but it would be a valuable contribution for surgeons. The idea is an excellent one.

We would like to thank the reviewer for this positive comment and feedback.

The article is also well written and with well-articulated objectives.

One paragraph still has me struggling to understand. Lines 159-165, where the optimization of the coordinates used in the CAS system were determined in the KneeKG system, in order to compare the two kinematic outputs. I don’t quite follow this, and I am wondering how a comparison can be made between passive knee flexion extension and gait.

We could have compared values in function of the flexion-extension angle measured with both methods. However, since we are interested in knee kinematics during daily life activities and specifically during gait, we chose to express the DoF measured during CAS in function of the gait cycle by using the knee flexion angle measured with KneeKG^TM^. This gives us a theoretical knee kinematics that would be equal to the knee kinematics measured with KneeKG if there were no differences between the two measurements.

We added a sentence in the section Data Analysis that clarifies the above comments:

L170: “Then, CAS kinematics were expressed as a gait cycle by matching the CAS knee flexion measurement with the corresponding average knee flexion angle measured using the KneeKG™ system during gait (Figure 2). In this way, the theoretical CAS kinematics during treadmill gait are determined and can be compared to the knee kinematics assessed with the KneeKG^TM^.”

Additionally, the optimisation procedure was slightly modified as follow for clarification:

L159: “The kinematic chain was introduced into the treadmill gait measurements using a two-level multi-body kinematics optimisation [13], where the variables to optimise were the fully Cartesian coordinates (uf, rPf, rDf, wf, ut, rPt, rDt, wt) and the positions of the reflective markers with respect to this kinematic chain. The outcome of the optimisation determined the optimal position of the kinematic chain, i.e. joint centres and flexion-extension axis, with respect to the thigh and shank clusters of the KneeKG™ system. These positions were used as the final calibration for the KneeKG™ system. In this way, the six degrees of freedom (DoF) kinematics were computed with the femur and tibia LCS matching those used during the CAS measurement.”

Line 170 – reference should be to figure 2, not figure 1

Thank you, this was modified accordingly in the manuscript.

Line 172 - How and why was the upsampling done?

Since we use the knee flexion angles to match the CAS and KneeKG^TM^ systems, we need to have as many values of the flexion angle similar between both measurements. We performed the upsampling to increase the number of correspondences between both measurements. Without the upsampling, the curves of the CAS expressed in percentage of the gait cycle had a significant number of occlusions. Upsampling was performed by using spline interpolation with the matlab function “ïnterp1”.

Line 174 – “mean AA angle, IE angle and AP displacement” – why these variables specifically? Does “mean” indicate that a mean angle was taken from the entire gait cycle, or from a specific knee flexion extension angle?

We used these three DoFs since they are the parameters reported in the original paper describing the KneeKG^TM^ system [1]. We assume that the medio-lateral displacement is too small to be accurately measured with the KneeKG^TM^ and the proximal-distal displacement only gives information on the shape of distal femur and proximal tibia but is not an actual DoF of the knee [2]. We added the reference to the paper by Hagemeister et al. in the paragraph, but, if the reviewer sees fit, we can add a sentence justifying this choice.

The mean was performed over the entire gait cycle for each DoF. This is why we have a range of values on figure 3 corresponding to 1SD of the measurement.

The paragraph was updated as follow:

L174: Each patient’s adduction–abduction (AA) angle, internal–external rotation (IER) angle and anterior–posterior (AP) displacement [5], as measured during the treadmill gait test were averaged over the gait cycles and compared using a Bland–Altman analysis [15] to the corresponding CAS measurements averaged over the gait cycles.

Line 190 – I think it would be helpful to understand if more detail were added to the caption for Figure 2, explaining in particular what data is shown in DoF = f(FE), how this is impacted by FE Gait, and again DoF in %GC.

The caption was updated as follow:

**Figure 2: Workflow of kinematic measurements and data analysis.** min f represents the two-level optimisation methods used to fit the calibration of the CAS system on the gait measurement, DoF = f(FE) represents the degrees of freedom measured with the CAS system expressed in function of the flexion-extension of knee, FE Gait represents the matching of CAS knee flexion-extension with knee flexion-extension measured during gait to obtain the DoF in percentage of GC (DoF in %GC).

Table 1 title – please define hka – hip knee ankle

This was modified accordingly in the manuscript.

I make the above suggestions since the value in this manuscript to readers is in understanding the methods the authors have used to relate the two distinctly derived knee kinematic measures. No other real conclusions can be drawn from this manuscript due to the low sample size, but this is appropriate for a preliminary study such as this one.

We fully agree with this comment and this was explained to reviewer #1.

**References:**

1.	Hagemeister N, Parent G, Van De Putte M, St-onge N, Duval N, de Guise J. A reproducible method for studying three-dimensional knee kinematics. Journal of biomechanics. 2005;38:1926-31. doi: 10.1016/j.jbiomech.2005.05.013.

2.	Gasparutto X, Moissenet F, Lafon Y, Chèze L, Dumas R. Kinematics of the normal knee during dynamic activities: a synthesis of data from intracortical pins and biplane imaging. Applied Bionics and Biomechanics. 2017;2017. doi: 10.1155/2017/1908618.
