# Supplementary material for: Comparison between passive knee kinematics during surgery and active knee kinematics during walking: A preliminary study
Source: PLoS One. 2023 Mar 6;18(3):e0282517. doi: 10.1371/journal.pone.0282517 (PMC9987822; doi:10.1371/journal.pone.0282517)
Supplement: Attachment — Submitted filename: NK - Response to reviewers - Review 2.docx [file pone.0282517.s004.docx]

Reviewer #3: This study sought to present a detailed comparison between passive knee kinematics (i.e., passive flexion-extension) acquired during total knee arthroplasty (TKA) by means of computer assisted-surgery system and active kinematics measured during walking with a marker-based optoelectronic system (KneeKG). The analysis was performed on 8 patients, who underwent TKA. Active kinematics was acquired before surgery and 3 months after, whereas passive kinematics was measured before and after TKA implantation. Anatomical axes were homogenised by using 2-levels optimization based on multi-body modelling, so as to limit any bias between active and passive kinematics. The authors compared knee joint angles acquired before and after TKA. The authors underlined very weak correlations between active and passive kinematics and they were not able to identify the source of errors.

General comment
The hypothesis at the basis of this paper is clearly reported as far as the main objective. Both the experimental phase and data analysis are written with a good level of details; the analysis in particular was performed well, and the synthesis of the obtained results is appreciable. Although the methodology in itself is not that innovative, the comparison provides useful hints and novel perspective for the application field.
The structure of the article seems to be precise (Abstract, Introduction, Methodology [with subheadings], Results, Discussion and Conclusions).
Experimental phase and data analysis seem to be clearly reported and are coherent with the work objectives. Several concerns have been reported to the authors.
The use of the English language seems to be correct.
Thank you for these kind comments.

Specific Comments
Title
Ok.

Abstract
In general, this section is quite ok.
Line 35-37: Please give few more hints concerning the “homogenization” technique.
The sentence was slightly lengthened to mention the reference calibration used:

L35: “The anatomical axes of the KneeKG™ and CAS systems were homogenised using a two-level, multi-body kinematics optimisation with a kinematic chain based on the calibration measured during CAS.”

Due to word count constraints (300 words in the abstract) more details were not reported.

Keywords
I would not use the name of a commercial system (KneeKG) as a keyword for a scientific work.
Introduction

“KneeKG” was removed from the keywords and replaced by Knee Harness.

Ok, well identified the main hypotheses and defined the main goal.
Thank you.

Materials and Methods
Ok, the experimental phase and data analysis was reported with a good level of detail.
Since the study was approved by an Ethical Committee, could you please give some information about the identification of the correct sample size? Did you perform any power analysis? How this led you to the possibility to generalize your results?

This study is a secondary analysis of a larger study aiming at characterising and understanding prosthetic knee instability in patients following total knee arthroplasty. The sample size calculation for the estimation (power 0.8, α 0.05) of a difference between stable and unstable patients was based on the primary outcome of knee anteroposterior movement (in mm) evaluated by an arthrometer on passive movement amplitudes as no data are available during walking. Mochizuki et al. (2017) reported an anteroposterior movement of the knee of approximately 8 ± 3.25 mm in patients after TKA with a sensation of instability compared to 6 ± 2.5 mm in patients with no sensation of instability. From these values, we computed a sample of 27 participants per group (27*2=54) (Figure 4) with a sample size calculator (G * Power software: Statistical Power Analyzes).

During this study, the recruitment and follow-up measurements were disturbed by the COVID situation and a change in the navigation system did not allow us to keep extracting the knee kinematics data for research purpose. These reasons led to this reduced sample size and to the preliminary nature of this study which was underlined in the manuscript and title of the paper.

This preliminary study allowed us to present a framework to compare both types of measurement and the first results of this comparison. We agree that a generalisation of the results cannot be performed with such population but our results can provide the basis for future studies and for power analysis evaluating the appropriate number of patients. Still, it seems reasonable to assume that, since we found a weak association between the two methods for 8 patients, it is very likely that there won’t be any association for more patients.

- Mochizuki, T., et al., Association between anteroposterior laxity in mid-range flexion and subjective healing of instability after total knee arthroplasty. Knee Surg Sports Traumatol Arthrosc, 2017. 25(11): p. 3543-3548.

The passive flexion-extension was performed while loading the limb from the foot? Did you think loads could influence your results?

As mentioned in the discussion: “the variability of the AA angle measured during pre-surgery treadmill gait was lower than those in the CAS measurements. This could suggest that muscle control under weight-bearing conditions can help to stabilise an arthritic knee joint. Interestingly, larger discontinuities and SDs in the AA angle and AP displacement were observed in the computer-assisted navigation data before TKA than after it. This may indicate that end-stage knee OA can lead to potential knee instability, as underlined by the greater variability in those two DoF. Post-TKA evaluations showed less variability and a more consistent pattern, which could indicate increased passive stability after TKA.”

The loads applied by the surgeon on the foot and shank may therefore influence the results, mainly on the AA and AP before definitive TKA implantation. A sentence has been added in this discussion section:

L 269: “This kinematics variability in joint kinematics also reflects the variability in the external loads magnitude and direction applied manually by the surgeons during passive flexion-extension.”

How did you identify the “gait cycle” over the passive
The “gait cycle” of the passive measurement represents the passive DoF of the knee over the knee flexion-extension range and pattern measured during gait. It was identified as the correspondence between the knee flexion-extension angle measured during gait and the knee flexion-extension angle during the passive movement. At each frame of the gait cycle, we identified the knee flexion-extension angle, then we found all frames in the passive flexion-extension where the knee flexion-extension angle was equal to the value measured during gait. At those frames, we selected the values of the different degrees of freedom of the knee and reported them as the “passive values” at this instant of the gait cycle.

Details were added to the explanation in the manuscript:

L 170: “Then, CAS kinematics were expressed as a gait cycle by matching the CAS knee flexion measurement with the corresponding average knee flexion angle measured using the KneeKG™ system during gait (Figure 2). The matching was performed in four steps: 1) at each frame of the treadmill gait cycle, the knee flexion-extension angle was identified, 2) all the frames from the CAS measurements with this knee flexion-extension angle were identified, 3) the values of the different degrees of freedom of the knee at those frames were identified, 4) those values were reported as the CAS values at this instant of the gait cycle.”

Results
Ok.

Discussion
Ok. Thank you for underlying the main limitations of your work.

Conclusions
Ok. My only concern is related to the possibility to generalize your results with such a small sample size.
As explained in the comment above, these results are not intended for generalisation.

References
The references to previous works seem to be precise, wide and up-to-date.
Thank you.

Figures
Ok.

Tables
Ok.
